# Whole-Genome Analysis of Human Papillomavirus Types 16, 18, and 58 Isolated from Cervical Precancer and Cancer Samples in Chinese Women

**DOI:** 10.1038/s41598-017-00364-9

**Published:** 2017-03-21

**Authors:** Ying Liu, Yaqi Pan, Weijiao Gao, Yang Ke, Zheming Lu

**Affiliations:** 1 Key Laboratory of Carcinogenesis and Translational Research (Ministry of Education), Laboratory of Genetics, Peking University Cancer Hospital & Institute, No. 52 Fucheng Road, Beijing, 100142 China; 2 Key Laboratory of Carcinogenesis and Translational Research (Ministry of Education), Department of Gynecologic Oncology, Peking University Cancer Hospital & Institute, No. 52 Fucheng Road, Beijing, 100142 China; 3 Key Laboratory of Carcinogenesis and Translational Research (Ministry of Education), Laboratory of Biochemistry and Molecular Biology, Peking University Cancer Hospital & Institute, No. 52 Fucheng Road, Beijing, 100142 China

## Abstract

Human papillomavirus (HPV) types 16, 18 and 58 are ranked the top three high-risk HPV types for cervical intraepithelial neoplasia (CIN) and invasive carcinoma. We aimed to evaluate the diversity of HPV16, HPV18, and HPV58 genetic variants by HPV capture technology combined with next generation sequencing. 295, 73, and 148 variations were observed in 51 HPV16, 7 HPV18, and 11 HPV58 genomes, respectively. HPV16 isolates were predominantly of the A variant lineage, and sublineage A4 (Asian) was the most common. However, there were no significant differences in the distribution of HPV16 A1–3 and A4 variants between CIN1-, CIN2/3, and cervical cancer groups. The 7 HPV18 genomes were assigned to the A3/A4 and A1 sublineages. Of the 11 HPV58 genomes, the most predominant variant sublineages were A2, followed by A1 and B2. The majority of HPV16/18 samples containing contiguous genomic deletions were found to harbor HPV integration. Some T-cell epitope sequences in HPV16 E6 and E7 showed considerable divergence from the prototype NC_001526, suggesting their importance in immunotherapy of HPV-associated carcinomas. In conclusion, sequence diversity and phylogenies of HPV16, 18, and 58 provide the basis for future studies of discrete viral evolution, epidemiology, pathogenicity, and the differences in response to vaccines.

## Introduction

Human papillomaviruses (HPVs) are a prevalent, globally distributed group of DNA viruses infecting cutaneous and mucosal epithelia throughout the human body^1^. HPVs contain a 7.9-kb circular double-stranded DNA genome that consists of four parts: an early region (E1, 2, 4–7 genes), a late region (L1, 2 genes), a long control region (LCR), and a small, highly variable, non-coding region (NCR) between E5 and L2. To date, more than 200 types of HPVs have been well characterized, and an individual HPV type is defined on the basis of the cloned genome being at least 10% different in the L1 open reading frame (ORF) nucleotide sequence from all other characterized HPV types^2^. HPV types 16, 18, and 58 are ranked the top three high-risk HPV types for cervical intraepithelial neoplasia (CIN) and invasive carcinoma and account for ~90% of all cervical cancers worldwide^3–5^.

Despite phylogenetic relatedness, HPV variants can differ in pathogenicity^6–9^. Most previous intratypic evolutionary studies of HPV variants used the partial regions of the viral genome that were generally limited to the E6, E7, and LCR regions through restriction enzyme polymorphisms and more recently through sequence determination of viral fragments^6, 8, 10, 11^. With the advent of next generation sequencing, HPV variant lineages and sublineages are now being investigated in great detail at the whole genome level. The use of multiple sequence alignments of complete viral genomes and phylogenetic analyses have begun to define variant lineages and sublineages using empirically defined differences of 1.0–10.0% and 0.5–1.0%, respectively^12^. Based on whole HPV genome sequencing, HPV type 16 can be divided into four main variant lineages (A/B/C/D) and nine sublineages: (1) A, including A1-A3 (previously named European) and A4 (Asian) sublineages; (2) B, including B1 (African-1, Afr1a) and B2 (African-1, Afr1b) sublineages; (3) C (African-2, Afr2a); and (4) D, including D1 (North American, NA1), D2 (Asian-American, AA2), and D3 (Asian-American, AA1) sublineages. HPV type 18 can be divided into three major lineages (A, B, and C) and additional sublineages (A1 to A5 and B1 to B3) that can be largely translated to the historical nomenclature (A1 and A2, = Asian American; A3 to A5, = European; and B/C, = African). HPV type 58 has 4 variant lineages (A/B/C/D) and 7 sublineages (A1 to A3, B1 to B2, and D1 to D2)^12^.

Recently, Mirabello L *et al.* ^7^ reported the largest study to date of HPV16 variant lineages and the risk of cervical precancer/cancer in 3200 women primarily from white, non-Hispanic population and first showed that the variant lineages that are often grouped are heterogeneous in pathogenicity. However, HPV16 variant lineages have co-evolved with specific human populations. Most women in their study had an HPV16 A1–3 variant lineage infection and only four cases of A4 sublineage^7^. Therefore, the relationship between HPV16 A4 sublineage and the risk of cervical precancer/cancer in Chinese populations living in China requires further study. To date, no studies on the role of HPV18 and HPV58 (sub)lineages in predicting cancer risk based on available complete genome sequences were reported, due to the whole-genome sequences of HPV16, HPV18, and HPV58 isolated from China being still limited.

Additionally, HPV integration essentially contributes to HPV-mediated neoplastic transformation and previous HPV variant studies lack high-resolution HPV breakpoints data. It is intriguing to determine which HPV variant lineage is preferentially integrated into the host genome. Recently a new method based on HPV-targeted hybrid capture together with flanking genomic sequences was described by our group^13^. Using this approach, HPV type, as well as variant and HPV integration in 47 tissue specimens with cervical cancer^13^ and 166 cervical biopsy specimens with normal cervical epithelium and CIN^14^ were analyzed, and 53 HPV16-, 12 HPV58-, and 7 HPV18-positive samples were identified.

In this study, variations of HPV16, HPV18, and HPV58 at the whole genome level were identified and the evolutionary phylogenies were described, aiming to provide basic data for future studies on their discrete evolution, epidemiology, pathogenicity and different responses to vaccines.

## Results

### Mutation and phylogenetic analysis of HPV16 sequences

Of the 53 HPV16-positive samples, 51 HPV16 genomes were assembled from CIN 1- (n = 8), CIN 2/3 (n = 15), and cervical cancer group (n = 28) (Supplementary Table S1). HPV16 genomes in T10 and T21 failed to be assembled, mainly due to the poor coverage across the genome. 43 of the 51 specimens successfully retrieved HPV16 full-length sequences, while 6 samples with cervical cancer and 2 samples with CIN 2/3 contained contiguous amplicons with very low or no sequence reads, which were interpreted as genome deletions. The deleted region often included fragments within the E2, E4, E5, and L2 genes, whereas the E6, E7, and LCR regions generated high sequence read numbers. Based on our previous HPV integration data^13, 14^, we confirmed that 5 of the 6 cervical cancer samples and 2 CIN 2/3 samples with deletions harbored HPV integration (Supplementary Table S1).

Compared with the HPV16 prototype reference (NC_001526), 295 variations in the 51 samples were observed (Table 1 and Supplementary Table S1), including 275 substitutions, 10 insertions, and 10 deletions. The proportion of nonsynonymous mutations in genes of E5, E6, L2, E2, and E4 was 53.85%, 57.14%, 58.33%, 82.50%, and 92.86% (Table 1).

**Table 1:** Calculation of HPV16, 18 and 58 variations by genome region and open reading frame (ORF).

Gene	Gene size (bp)	Point mutation (N)							Deletion (N)	Insertion (N)	Total	Variation rate
		Total	Non-synonymous mutations			Synonymous mutations						
			Total	Ts	Tv	Total	Ts	Tv				
**HPV16 (n = 51)**												
E6	477(83–559)	14	8(57.14%)	5	3	6(42.86%)	3	3	0	0	14	2.93%
E7	297(562–858)	9	2(22.22%)	2	0	7(77.78%)	6	1	0	0	9	3.03%
E1	1949(865–2813)	45	17(37.78%)	7	10	28(62.22%)	22	6	0	1	46	2.36%
E2	1098(2755–3852)	40	33(82.50%)	18	15	7(17.50%)	4	3	0	0	40	3.64%
E4	288(3332–3619)	14	13(92.86%)	8	5	1(7.14%)	1	0	0	0	14	4.86%
E5	252(3849–4100)	13	7(53.85%)	3	4	6(46.15%)	4	2	0	0	13	5.16%
L2	1422(4236–5657)	60	35(58.33%)	17	18	25(41.67%)	17	8	3	0	63	4.43%
L1	1596(5560–7155)	35	14(40.00%)	5	9	21(60.00%)	17	4	3	1	39	2.44%
LCR	832(7156–7905 1–82)	43	—	—	—	—	—	—	1	2	46	5.53%
NCR	135(4101–4235)	18	—	—	—	—	—	—	5	6	29	21.48%
**HPV18 (n = 7)**												
E6	477(105–581)	3	0(0.00%)	0	0	3(100.00%)	1	2	0	0	3	0.63%
E7	318(590–907)	2	1(50.00%)	1	0	1(50.00%)	1	0	0	0	2	0.63%
E1	1974(914–2887)	11	3(27.27%)	1	2	8(72.73%)	6	2	1	1	13	0.66%
E2	1098(2817–3914)	11	6(54.54%)	1	5	5(45.45%)	3	2	1	1	13	1.18%
E4	267(3418–3684)	3	3(100.00%)	1	2	0(0.00%)	0	0	0	0	3	1.12%
E5	222(3936–4157)	0	0(0.00%)	0	0	0(0.00%)	0	0	0	0	0	0.00%
L2	1389(4244–5632)	17	9(52.94%)	4	5	8(47.06%)	2	6	0	0	17	1.22%
L1	1707(5430–7136)	17	9(52.94%)	3	6	8(47.06%)	4	4	0	0	17	0.99%
LCR	825(7137–7857 1–104)	9	—	—	—	—	—	—	0	0	9	1.09%
NCR	107(3915–3935 4158–4243)	4	—	—	—	—	—	—	0	0	4	3.74%
**HPV58** (**n = 11**)												
E6	450(110–559)	3	2(66.67%)	0	2	1(33.33%)	1	0	0	0	3	0.67%
E7	297(574–870)	10	5(50.00%)	4	1	5(50.00%)	4	1	0	0	10	3.37%
E1	1935(883–2817)	20	7(35.00%)	0	7	13(65.00%)	11	2	1	0	21	1.08%
E2	1077(2753–3829)	16	10(62.50%)	3	7	6(37.50%)	3	3	0	0	16	1.48%
E4	276(3330–3605)	9	9(100.00%)	3	6	0(0.00%)	0	0	0	0	9	3.26%
E5	231(3892–4122)	9	0(0.00%)	0	0	9(100.00%)	6	3	0	0	9	3.90%
L2	1419(4244–5662)	31	7(22.58%)	2	5	24(77.42%)	15	9	0	0	31	2.18%
L1	1575(5565–7139)	30	10(33.33%)	8	2	20(66.67%)	16	4	0	0	30	1.90%
LCR	794(7140–7824 1–109)	21	—	—	—	—	—	—	0	0	21	2.64%
NCR	121(4123–4243)	6	—	—	—	—	—	—	0	1	7	5.78%

Abbreviations: Ts, Transition; Tv, Transversion.

295 variations were shown to be broadly distributed across the whole genome, including E6 (n = 14), E7 (n = 9), E1 (n = 46), E2 (n = 25), E2/E4 (n = 14), E2/E5 (n = 1), E5 (n = 12), L2 (n = 60), L2/L1 (n = 3), L1 (n = 36), LCR (n = 46), and NCR (n = 29) (Table 1). Taking into consideration the different length of each fragment (i.e., number of single nucleotide polymorphism (SNPs)/region size in nucleotides), the most common variant regions were NCR (21.48%, 29/135), followed by LCR (5.53%, 46/832), E5 (5.16%, 13/252), E4 (4.86%, 14/288), L2 (4.43%, 63/1422), E2 (3.64%, 40/1098), E7 (3.03%, 9/297), and E6 (2.93%, 14/477), and the sparsest were L1 (2.44%, 39/1596), and E1 (2.36%, 46/1949). The genome sizes of HPV16 sequence, estimated based on the reference HPV16 genome (NC_001526), were ranging from 7770 bp to 7909 bp, mainly due to the insertions and deletions (indels). A 135-bp deletion within the L2/L1 overlap region (positions 5614–5748 del) in CIN 3–6 (7770 bp in length) was observed. A 30-bp deletion within the L2 ORF (positions 4622–4651 del) was found in CIN 3–3 (7875 bp in length). Except for 3 indels that occurred in all the 51 HPV genomes, other indels in CIN 1–34 (7886 bp in length) were within the highly variable non-coding region between E5 and E2 (NCR, positions 4101–4235) of the HPV16 genome (Supplementary Table S1).

**Figure 1:**
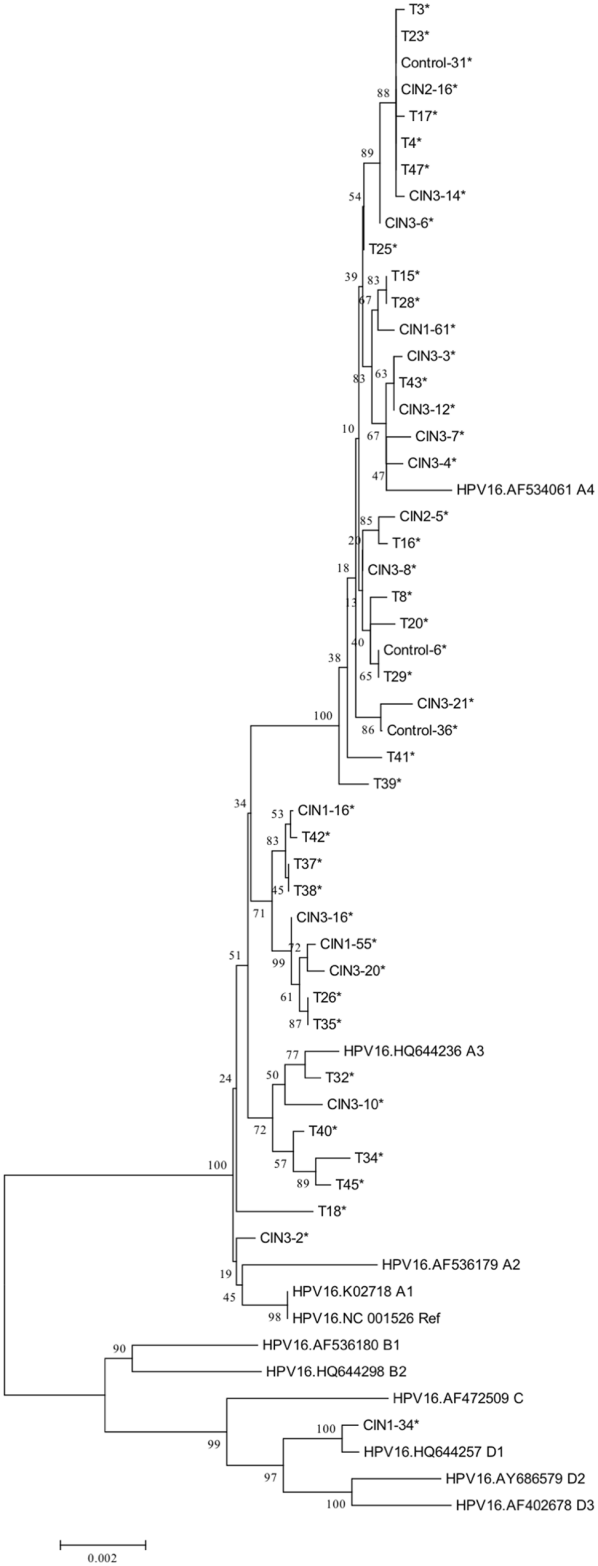
Phylogenetic tree based on whole-genome analyses of 46 HPV16-positive samples and 11 sequences from the Genbank database. Phylogenetic analyses were conducted using the neighbor-joining algorithm implemented in MEGA software (version 6). Bootstrap analysis of 1000 replicates was performed on each tree to determine the confidence. Study sequences in the phylogenetic trees were marked with an asterisk.

The same sequences in Control-30 and T4, T5 and T8, T29, T33 and T36, CIN 2–11 and T38 were observed, thus 46 unique HPV16 genomes were obtained. The phylogenetic analysis was conducted according to multiple nucleotide sequence alignments of whole genomes, including the unique 46 HPV16 genomes identified in this study and 11 known representative HPV16 variant lineages and sublineages (for NCBI accession numbers see Fig. 1). Analyses on whole HPV16 genomes indicated that 29 viral strains were closely related to HPV16 A4 strain, 16 viral strains were closely related to HPV16 A1–A3 strains, and 1 viral strain was closely related to HPV16 D1 strain (Fig. 1). Thus, among the 51 HPV16-positive samples, the most predominant variant sublineages were A4 (Asian) (64.70%, 33/51), followed by A1–3 (European) (33.33%, 17/51) and D1 (North American) (1.96%, 1/51).

### Mutation and phylogenetic analysis of HPV18 sequences

Seven HPV18 genome sequences were obtained from CIN 1- (n = 3) and cervical cancer groups (n = 4) (Supplementary Table S2). The 3 isolates from CIN 1- samples generated complete genome sequences, while the 4 isolates from cervical cancer samples contained contiguous amplicons with very low or no sequence reads, which were interpreted as genome deletions. The deleted region often included fragments within the E2, E4, E5, L2, and L1 genes, whereas the E6, E7, and LCR regions generated high sequence read numbers. On the basis of validated HPV integration breakpoints in our previous study^13^, we found that 3 of the 4 cervical cancer samples with deletions harbored HPV integration (Supplementary Table S2).

In comparison to the reference HPV18 genome (NC_001357), 7 HPV18 genomes harbored 73 variations (0.93% of genome), including 71 substitutions, 1 insertion, and 1 deletion. The 73 variations were found to be located on virtually all genome except the E5 gene, including E6 (n = 3), E7 (n = 2), E1 (n = 11), E1/E2 (n = 2), E2 (n = 8), E2/E4 (n = 3), L2 (n = 14), L2/L1 (n = 3), L1 (n = 14), LCR (n = 9), and NCR (n = 4) (Table 1).

**Figure 2:**
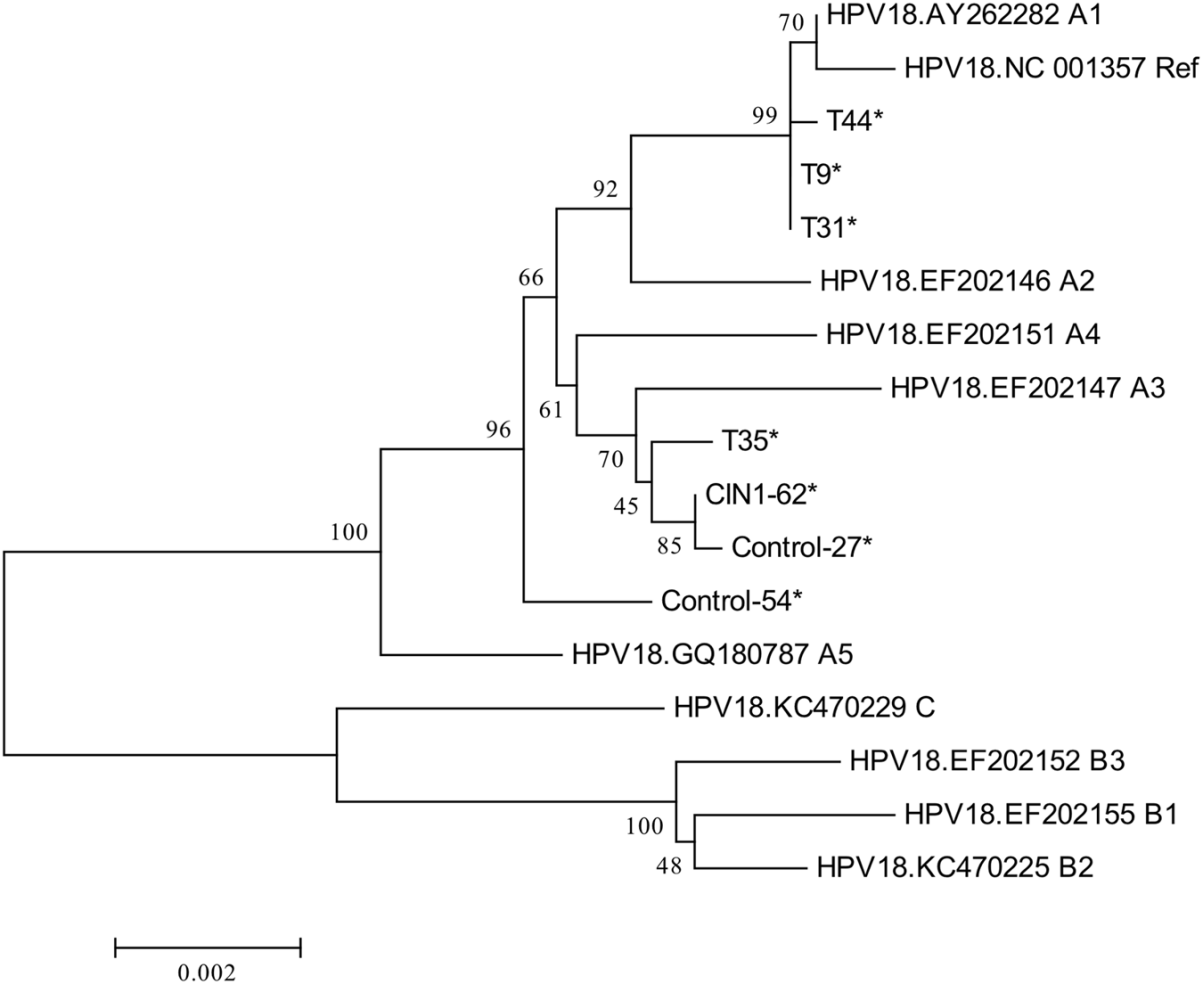
Phylogenetic tree based on whole-genome analyses of 7 HPV18-positive samples and 10 published representative HPV18 variant lineages and sublineages from the Genbank database. Phylogenetic analyses were conducted using the neighbor-joining algorithm implemented in MEGA software (version 6). Bootstrap analysis of 1000 replicates was performed on each tree to determine the confidence. Study sequences in the phylogenetic trees were marked with an asterisk.

The phylogenetic analysis was conducted based on multiple nucleotide sequence alignments of whole genomes, including the unique 7 HPV18 genomes in this study and the 10 published representative HPV18 genomes for viral variant sublineages. Analyses on whole HPV18 genomes indicated that all the 7 viral strains were mapped to the HPV18 A lineage (Fig. 2). Of the 7 HPV18-positive samples, the predominant variant sublineages were A3/A4 (57.14%, 4/7), followed by A1 (42.86%, 3/7).

### Mutation and phylogenetic analysis of HPV58 sequences

Of the 12 HPV58-positive samples, 11 HPV58 genomes were assembled from CIN 1- (n = 4), CIN 2/3 (n = 3), and the cervical cancer group (n = 4) (Supplementary Table S3). HPV58 genome in T9 failed to be assembled, mainly due to the low level of the average sequencing depth on target. All the 11 specimens achieved HPV58 full-length sequences. The genome sizes of HPV58 ranged from 7821 bp to 7825 bp.

In comparison to the reference HPV58 genome (D90400), the 11 samples harbored 148 variations (1.89% of genome), including 146 substitutions, 1 insertion and 1 deletion (Table 1). The 148 variations were shown to be broadly distributed across the whole genome, including E6 (n = 3), E7 (n = 10), E1 (n = 21), E2 (n = 7), E2/E4 (n = 9), E5 (n = 9), L2 (n = 31), L1 (n = 30), LCR (n = 21), and NCR (n = 7) (Table 1).

**Figure 3:**
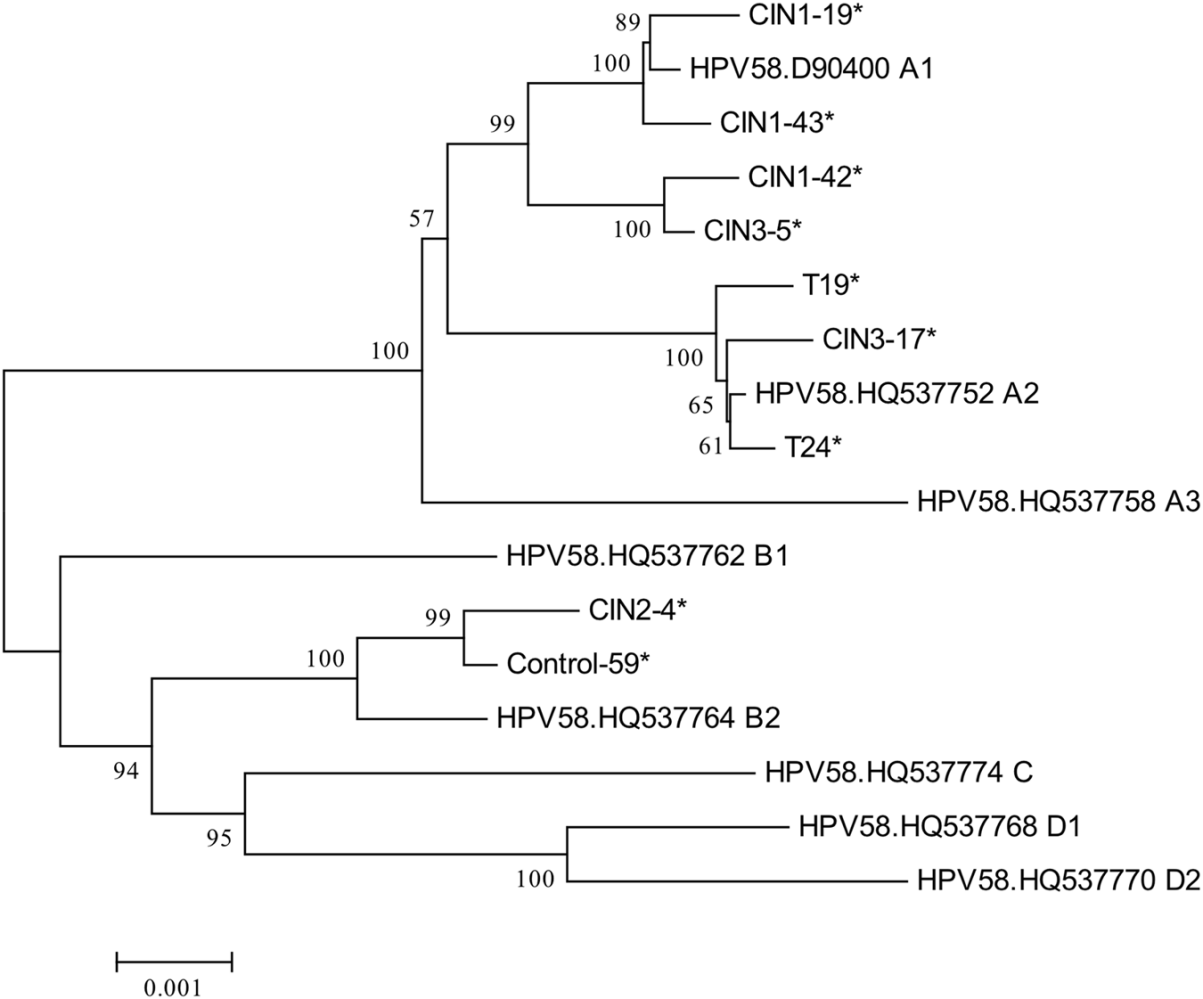
Phylogenetic tree based on whole-genome analyses of 9 HPV58-positive samples and 8 published representative HPV58 variant lineages and sublineages from the Genbank database. Phylogenetic analyses were conducted using the neighbor-joining algorithm implemented in MEGA software (version 6). Bootstrap analysis of 1000 replicates was performed on each tree to determine the confidence. Study sequences in the phylogenetic trees were marked with an asterisk.

The same sequences in T11, T19, and T30 were observed, therefore, 9 unique HPV58 genomes were obtained. The phylogenetic analysis was conducted based on multiple nucleotide sequence alignments of whole genomes, including the 9 newly available unique HPV58 genomes in this study and 8 previously published representative HPV58 genomes for viral variant lineages and sublineages. Analyses on whole HPV58 genomes indicated that 7 viral strains were closely related to HPV58 lineage A and 2 viral strains were closely related to HPV58 lineage B (Fig. 3). Of the 11 HPV58-positive samples, the most predominant variant lineages were A2 (45.45%, 5/11), followed by A1 (36.36%, 4/11), and B2 (18.18%, 2/11) (Supplementary Table S3).

### Association of HPV16 variants with cervical cancer risk and HPV integration

Because of the smaller number of samples with HPV18 and HPV58 genomes, only the association between HPV16 variants and the risk of cervical precancer/cancer was analyzed. 51 HPV16-positive samples were grouped into CIN 1-, CIN 2/3, and cervical cancer groups. Most women in our study (50/51, 98.04%) had an HPV16 A variant lineage infection, including 17 A1–3 (33.33%) and 33 A4 (64.71%), with 2 A1–3 and 5 A4 in CIN 1-, 5 A1–3 and 10 A4 in CIN 2/3, and 10 A1–3 and 18 A4 in the cervical cancer group. There was only one woman (1.96%) with an HPV16 sublineage D1 infection in the CIN 1- group. Thus, we assessed the association of HPV16 sublineage A4 (Asian) with cervical precancer and cancer in comparison of the sublineages A1–3 (European). No significant difference in the distribution of A1–3 and A4 variants was observed between CIN 1-, CIN 2/3, and cervical cancer groups (*P* = 0.936) (Table 2).

**Table 2:** Association between HPV16 variants and cervical cancer.

Variant	CIN 1-	CIN 2/3	Cancer	*P*
A1–A3	2	5	10	0.936
A4	5	10	18	

To determine which HPV variant lineage is preferentially integrated into the host genome, the ratio of integration in different HPV 16 variant sublineages was investigated. Out of the 17 HPV16 A1–3 isolates, 47% (8/17) were integrated into the host genome. Of the 33 HPV16 A4 isolates, 48% (16/33) were integrated into the host genome (Table 3).

**Table 3:** Association between HPV16 A variants and HPV16 integration.

Variant	HPV integration		*P*
	Yes	No	
A1–A3	8	9	0.924
A4	16	17	

### Amino acid changes in CD4^+^ and CD8^+^ T-cell epitopes of HPV16 E6 and E7

**Figure 4:**
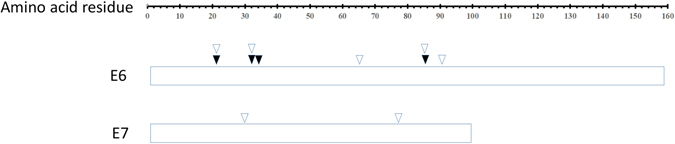
Amino acid changes in CD4^+^ and CD8^+^ specific T cell epitopes of HPV16 E6 and E7 proteins. Amino acid changes in at least one of the 51 HPV16 isolates at CD4^+^ and CD8^+^ specific T cell epitopes are marked with hollow and solid arrows, respectively. Stacking arrows indicate that the amino acid change is in a peptide which serves as both CD4^+^ and CD8^+^ epitopes.

The extensive genetic diversity within HPV16 E6 and E7 can have a significant impact on immune recognition of these antigens. A comprehensive investigation into the immunological impact of HPV16 E6 and E7 sequence polymorphism is essential for optimizing the adoptive cell therapy approaches and vaccine development strategies. According to the epitopes specific for both CD4^+^ and CD8^+^ T cells defined and reviewed in previous publications^15–22^, amino acid changes were found in 5 CD4^+^ epitopes and 5 CD8^+^ epitopes of HPV16 E6 protein, 5 CD4^+^ epitopes of HPV16 E7 protein (Supplementary Tables S4 and S5). Some of the nonsynonymous mutations were affecting multiple epitopes. For example, a C-to-T substitution at coordinate 790 (NC_001526) resulted in the change of residue 77 (R → C) in HPV16 E7 in only one HPV16 isolate, where CD4^+^ epitopes GQA (E7 43–77), CDS (E7 61–80), STH (E7 71–85), and IRT (E7 76–86) were located. A G-to-A substitution at coordinate 176 resulted in the change of residue 32 (D → N) in HPV16 E6 in 4 HPV16 isolates, a T-to-G substitution at coordinate 178 resulted in the change of residue 32 (D → E) in HPV16 E6 in 33 HPV16 isolates, and a T-to-C substitution at coordinate 183 resulted in the change of residue 34 (I → T) in HPV16 E6 in only 1 HPV16 isolate, where CD8^+^ epitopes TIH (E6 29–37), TIH (E6 29–38), and HDI (E6 31–38) were located. The positions of the nonsynonymous changes located in the epitopes are illustrated in Fig. 4 and tabulated in Supplementary Tables S4 and S5.

## Discussion

It was well recognized that the association of HPV and cervical carcinogenesis was based on the presence of HPV DNA sequence^23^. With the development and improvement of assays for HPV detection and analysis, the underlying nucleotide changes could be comprehensively studied. Here, we adopted HPV capture technology combined with next generation sequencing not only to detect the complete genome sequences, but also to identify HPV integration. In the current study, *de novo* assembly was successfully performed for 69 HPV genomes, including 51 HPV16 genomes, 7 HPV18 genomes, and 11 HPV58 genomes. We described the genome variability and evolutionary phylogeny of HPV16, HPV18, and HPV58. Our whole genome sequence-based data enabled us to make several novel observations that could not be available with sequencing of limited regions. With this dataset, we were able to find more SNPs and define HPV lineages and sublineages across the genome.

The diversity of the ORFs/regions varies by HPV types. Each ORF/region differs in sequence diversity, for HPV16, from most variable to least variable: NCR > LCR/E5 > E4/L2 > E2/E7/E6 > L1/E1; for HPV18, NCR > L2/E2/E4 > LCR/L1 > E1/E6/E7; for HPV58, NCR > E5/E7/E4 > LCR/L2 > L1/E2 > E1/E6. Our observation supported that L1 is a conserved region of HPV; however, there are still a number of SNPs which may affect the efficacy of vaccination to some extent. It is worth noting that, both Cervarix^®^ and Gardasil^®^ are virus-like particles (VLPs) of L1, which are licensed for use as prophylactic vaccines for HPV in many countries worldwide. Our work showed genetic variability of L1, making it essential to take into account the HPV16 variant lineages or population stratification when developing vaccines^24^.

It is well known that HPV16 variant lineages have co-evolved with specific human populations and are most prevalent in specific geographic regions. Our results confirmed that A4 lineages account for the majority of HPV16 isolates obtained from Chinese populations. Compared with women with an HPV16 A1–A3 European lineage infection, women with an HPV16 A4 Asian lineage infection had no significantly increased risk for cervical cancer. However, Hang *et al.* reported that A4 variants appear to predict higher risk of cervical cancer among HPV16-positive women^25^. Thus, further study is required to determine whether A4/Asian variants represent a higher risk for cervical cancer. Additionally, functional studies regarding HPV polymorphisms across the HPV16 genome of A4/Asian variants should be conducted to explore the biological evidence of carcinogenicity.

Some previous studies investigating HPV16 full length sequences in cervical specimens have shown that the contiguous deletions identified to be highly associated with cancer are suggestive of a pattern of HPV integration^26, 27^. However, HPV integration breakpoints were not available due to the limitation of the methods in these studies. Through analyzing the corresponding HPV integration breakpoints validated via Sanger sequencing in our previous study^13^, 87.5% (7/8) of the 8 HPV16 samples and 75.0% (3/4) of the 4 HPV18 samples containing contiguous genomic deletions were found to harbor HPV integration, indicating that these deletions are indeed signatures of HPV integration. HPV DNA can integrate into the host genome, sometimes in precancer and especially in cancers^28^. The deletion patterns observed in our study indicated that the HPV16/18 DNA in the specimens with CIN 2/3 or cervical cancer may exist in the pure integrated form. Moreover, out of the retrieved 43 full-length HPV16 specimens, 39.5% (17/43) were also found to harbor HPV integration breakpoints broadly distributed across the whole genome (Supplementary Table S1), revealing a mixture of episomal and integrated forms in these samples. It is of significant interest to observe that small deletions of 177 to 706 bp in size for HPV16 and a 24-bp deletion for HPV18 failed to be amplified by PCR amplification, which further validated the hypothesis that these deletions are due to the HPV DNA integration into the host genome in these samples. On the contrary, a 135-bp deletion in CIN 3–6 (7770 bp in length) and a 30-bp deletion in CIN 3–3 (7875 bp in length) existed in biopsies and were confirmed by PCR amplification and Sanger sequencing. This minor region of HPV deletion has not been reported in HPV genomes before.

The HPV-encoded early proteins, especially oncoproteins E6 and E7, are promising tumor-specific antigens to be targeted by immunotherapeutic approach since they are consistently expressed in HPV-associated malignancies and precancerous lesions. Indeed, adoptive transfer of E6/E7-targeted T cells is able to induce regression of cervical cancer^29, 30^. In this study, sequence analysis of the gene encoding E6 and E7 in HPV isolates from 51 HPV-positive specimens has revealed considerable *E6* and *E7* sequence divergence from the prototype NC_001526. Importantly, T cells recognition of E6 and E7 epitopes might be greatly affected by this sequence polymorphism, which implies that HPV variant-specific epitopes are warranted to expand and activate functional adoptive T cells.

Further studies with complete sequencing of HPV genomes from large population-based and case-control studies of cervical precancer and cancer are required to understand viral carcinogenesis and possibly to improve preventive and therapeutic strategies in the future.

## Materials and Methods

### Study population and specimen collection

A total of 47 fresh tissue specimens were collected from patients with cervical cancers who had undergone surgeries at Anyang Cancer Hospital, Henan province, China, between 2009 and 2010. A total of 166 cervical biopsy specimens were collected and diagnosed with CIN 1- (including normal cervical epithelium or acute/chronic cervicitis without atypical hyperplasia [n = 64] and CIN 1 [n = 62]) and CIN 2/3 (including CIN 2 [n = 19] and CIN 3 [n = 21]) from Beijing Cancer Hospital, Beijing, China, between 2014 and 2015.

Individual informed consent had been collected from all study participants. This study received ethical approval from the Institutional Review Board of both hospitals. All experiments were performed in accordance with relevant guidelines and regulations.

### DNA preparation

DNA was extracted using DNeasy Blood & Tissue kit (Qiagen, Hilden, Germany) according to the manufacturer’s protocol. DNA concentration was determined via a Nano-Drop (NanoDrop Technologies, Wilmington, DE, USA).

### HPV typing

The overall process of HPV capture and sequencing was previously described^13, 14^. HPV probes were designed according to the full-length genome of 17 HPV types (6, 11, 16, 18, 31, 33, 35, 39, 45, 52, 56, 58, 59, 66, 68, 69, and 82) by MyGenostics (MyGenostics, Baltimore, MD, USA).

HPV typing was reported in our previous study^13, 14^. Taking into consideration the sample number of the same HPV type, only HPV16, HPV18, and HPV58-positive samples were analyzed in subsequent HPV assays, i.e., 53 HPV16-, 12 HPV58-, and 7 HPV18-positive samples were included.

### *De novo* assembly of HPV genomes

After filtering low quality reads and trimming 3′/5′ adaptors, HPV-paired clean reads were assembled using Velvet 1.2.10 (with parameters −ins_length 180, −exp_cov auto). The settings were optimized for each sample using k-mer lengths of 59–73. Then the contigs were blasted against the NCBI nt database and the contigs mapped to the human genome were removed. The location and orientation of contigs were evaluated by pairwise alignment of the contigs to the reference HPV genome. The gaps between the contigs were linked up using either Sanger sequencing or tracts of “N” with length estimated based on the reference HPV genome.

### Mutation analysis

Illumina clean reads were mapped to reference HPV genome using the BWA program (HPV16 reference genome NC_001526, HPV18 reference genome NC_001357, HPV58 reference genome D90400). The quality scores were recalibrated and realigned to reference sequences using the GATK software package. Single nucleotide variants (SNVs) and insertion and deletions (indels) were detected and genotyped with the GATK HaplotypeCaller in single-sample mode. SNVs and indels were filtered with GATK VariantFiltration module (with filters “QUAL < 50.0 & QD < 1.5 & FS > 10 & DP < 5”). Substitutions, insertions, and deletions were all considered as variations. Positions marked with “N” were ignored in the mutation analysis.

### PCR amplification and Sanger sequencing

To validate the HPV variant sites, we designed primers to amplify regions of interest by PCR. PCR products were sequenced directly from both directions using conventional Sanger sequencing. All sequences were blasted by the NCBI human mega Blast database alignment tool. PCR primers were shown in Supplementary Table S6.

### Phylogenetic analysis of HPV16, HPV18, and HPV58 genomes

Phylogenetic analysis was performed using Molecular Evolutionary Genetics Analysis version 6 (MEGA6) and the neighbor-joining algorithm. This approach was based on multiple sequence alignments of the whole genomes of HPV16, HPV18, and HPV58. Bootstrap analysis of 1000 replicates was performed on each tree to determine the confidence.

### Statistical analysis

Fisher’s exact test was used for statistical analysis in the present study. *P*-value of less than 0.05 was considered as statistically significant. All the *P*-values presented are two-sided.

## Electronic supplementary material

Supplementary Tables
