# Supplementary material for: Whole-Genome Analysis of Human Papillomavirus Types 16, 18, and 58 Isolated from Cervical Precancer and Cancer Samples in Chinese Women
Source: Sci Rep. 2017 Mar 21;7:263. doi: 10.1038/s41598-017-00364-9 (PMC5428204; doi:10.1038/s41598-017-00364-9)
Supplement: Supplementary file 1 — Supplementary Tables [file 41598_2017_364_MOESM1_ESM.doc]

**Title:**

**Whole-Genome Analysis of Human Papillomavirus Types 16, 18, and 58 Isolated from Cervical Precancer and Cancer Samples in Chinese Women**

Ying Liu^1^, Yaqi Pan^1^, Weijiao Gao^2^, Yang Ke^1*^, Zheming Lu^3*^

Supplementary Table S1. Genomic variations of 51 HPV16-positive samples

Supplementary Table S2. Genomic variations of 7 HPV18-positive samples

Supplementary Table S3. Genomic variations of 11 HPV58-positive samples

Supplementary Table S4. CD4 T-cell epitopes of HPV-16 E6 and E7 proteins described, with HLA restriction elements identified

Supplementary Table S5. CD8 T-cell epitopes of HPV-16 E6 and E7 proteins described, with HLA restriction elements identified

| Supplementary Table S6. The PCR primers for validating the HPV variations | | | |
| --- | --- | --- | --- |
| Primer name | | Primer sequence 5'-3' | PCR Product (bp) |
|  | HPV16F7336-7359 | caacacctactaattgtgttgtgg | 649 |
|  | HPV16R58-79 | atgtctgcttttatactaaccg |
|  | HPV16F45-64 | gttgaaccgaaaccggttag | 589 |
|  | HPV16R613-633 | acagtagagatcagttgtctc |
|  | HPV16F562-581 | atgcatggagatacacctac | 559 |
|  | HPV16R1099-1120 | gaacctgtactgcatctctatg |
|  | HPV16F1098-1117 | acatagagatgcagtacagg | 503 |
|  | HPV16R1581-1600 | ctgggtgtaagtccaaatgc |
|  | HPV16F1554-1573 | tcaacgtgttgcgattggtg | 630 |
|  | HPV16R2163-2183 | cttccaatcacctccatcatc |
|  | HPV16F2161-2180 | tagatgatggaggtgattgg | 633 |
|  | HPV16R2773-2793 | gtcctgacacacatttaaacg |
|  | HPV16F2570-2590 | tgctggtacagattctaggtg | 477 |
|  | HPV16R3027-3047 | ctaacgtcttgtaatgtccac |
|  | HPV16F2753-2773 | cgatggagactctttgccaac | 606 |
|  | HPV16R3338-3358 | tgctaaacacagatgtaggac |
|  | HPV16F3173-3193 | gtgaagaagcatcagtaactg | 537 |
|  | HPV16R3692-3710 | catgtagacgacactgcag |
|  | HPV16F3322-3344 | ggtcaggtaatattatgtcctac | 650 |
|  | HPV16R3950-3971 | tgatgtgtatgtagacacagac |
|  | HPV16F3602-3622 | gtaacactacacccatagtac | 690 |
|  | HPV16R4272-4292 | ataaagttgggtagccgatgc |
|  | HPV16F3934-3952 | tacgtccgctgcttttgtc | 582 |
|  | HPV16R4494-4515 | ttaaagggggtcttacaggagc |
|  | HPV16F3917-3937 | gtgtgtctgcctattaatacg | 682 |
|  | HPV16R4581-4599 | atgttggtgcaccagcatc |
|  | HPV16F4475-4495 | cacagctacagatacacttgc | 773 |
|  | HPV16R5227-5247 | cttctgcaggatcaatagtac |
|  | HPV16F4495-4516 | ctcctgtaagaccccctttaac | 645 |
|  | HPV16R5120-5139 | taatgccagtacgcctagag |
|  | HPV16F5093-5113 | agttgctttacataggccagc | 583 |
|  | HPV16R5657-5675 | aagtagacagtggcctcac |
|  | HPV16F5519-5538 | tcctatagttccagggtctc | 332 |
|  | HPV16R5830-5851 | ccctgtattgtaatcctgatac |
|  | HPV16F5634-5653 | tcagatgtctctttggctgc | 639 |
|  | HPV16R6253-6272 | gtagtaaagtccatagcacc |
|  | HPV16F6248-6269 | gctttggtgctatggactttac | 560 |
|  | HPV16R6788-6807 | gtatgtcataacgtctgcag |
|  | HPV16F6729-6748 | acgacatggggaggaatatg | 631 |
|  | HPV16R7339-7359 | ccacaacacaattagtaggtg |
|  |  |  |  |
|  | HPV18F7293-7312 | gtggttctgtgtgttatgtg | 717 |
|  | HPV18R133-152 | atcaggtagcttgtagggtc |
|  | HPV18F139-158 | acaagctacctgatctgtgc | 694 |
|  | HPV18R812-832 | gtctgctgagctttctactac |
|  | HPV18F808-828 | gctagtagtagaaagctcagc | 574 |
|  | HPV18R1362-1381 | gccatgttcgccatttgtag |
|  | HPV18F1718-1738 | gcccatattcaatgtctagac | 573 |
|  | HPV18R2272-2290 | ctctatttgttggtatcgc |
|  | HPV18F2245-2264 | agattggagaccaatagtgc | 677 |
|  | HPV18R2901-2921 | ctgtatttggctgtctatgtc |
|  | HPV18F2861-2880 | gcaggacaaaatcatagacc | 614 |
|  | HPV18R3454-3474 | gtttaacaagctgagtagcgg |
|  | HPV18F3419-3439 | tgactctatgtgcagtaccag | 622 |
|  | HPV18R4021-4040 | caatacccatgcatacgcac |
|  | HPV18F3967-3986 | gtgtatgcatgtatgtgtgc | 646 |
|  | HPV18R4592-4612 | agtaaacgtaggcctaggtgc |
|  | HPV18F4565-4584 | gaggactccagtgtggttac | 606 |
|  | HPV18R5151-5170 | gctgcgggtaaacatagttg |
|  | HPV18F5123-5144 | gttcgctttagtagattaggtc | 651 |
|  | HPV18R5754-5773 | gcaggaaccctaaaatatgg |
|  | HPV18F5715-5734 | tatcatgctggcagctctag | 625 |
|  | HPV18R6318-6339 | cataaggatctgcagacatttg |
|  | HPV18F6231-6252 | gccatggactttagtacattgc | 602 |
|  | HPV18R6813-6832 | acaccaaagttccaatcctc |
|  | HPV18F6670-6689 | ctgggcaatatgatgctacc | 643 |
|  | HPV18R7293-7312 | cacataacacacagaaccac |
|  |  |  |  |
|  | HPV58F7749-7770 | ctcactaacatttattgccagg | 640 |
|  | HPV58R539-560 | gttacacttgtgtttgtctacg |
|  | HPV58F549-571 | cacaagtgtaacctgtaacaacg | 636 |
|  | HPV58R1166-1184 | acacagtcctctacagcac |
|  | HPV58F1123-1142 | gctgtgtgtgcactaaaacg | 554 |
|  | HPV58R1658-1676 | cctctgtcacacgttaaac |
|  | HPV58F1657-1676 | tgtttaacgtgtgacagagg | 527 |
|  | HPV58R2164-2183 | ggtctccaattacctccatc |
|  | HPV58F2164-2183 | gatggaggtaattggagacc | 608 |
|  | HPV58R2751-2771 | gtgctgatatttcctccatcg |
|  | HPV58F2907-2926 | cacatttgtgccaccaggtg | 643 |
|  | HPV58R3528-3549 | cagttagttgtactgtgtagtc |
|  | HPV58F3528-3549 | gactacacagtacaactaactg | 592 |
|  | HPV58R4096-4119 | gtcttgttgggttaagtattgtgc |
|  | HPV58F4096-4119 | gcacaatacttaacccaacaagac | 647 |
|  | HPV58R4724-4742 | atggctcagtaaaggaggg |
|  | HPV58F4853-4873 | tctactgacagtggcaatgtc | 608 |
|  | HPV58R5442-5460 | agaggagtgtcaaatccag |
|  | HPV58F5441-5459 | actggatttgacactcctc | 604 |
|  | HPV58R6024-6044 | gttactggtttcagtgtcatc |
|  | HPV58F6179-6198 | tgcagctgctactgattgtc | 566 |
|  | HPV58R6722-6744 | cttcaacatgacgtacatattcc |
|  | HPV58F6722-6744 | ggaatatgtacgtcatgttgaag | 634 |
|  | HPV58R7332-7355 | ccttactcatagatacacccaaac |
|  | HPV58F7336-7355 | gggtgtatctatgagtaagg | 435 |
|  | HPV58R7749-7770 | cctggcaataaatgttagtgag |
